# Maternal infection of SARS-CoV-2 during the first and second trimesters leads to newborn telomere shortening

**DOI:** 10.1186/s12967-024-05879-0

**Published:** 2024-11-21

**Authors:** Lina Wang, Junfeng Zhang, Fangfei Liu, Qixiang Shi, Fengchun Gao, Junmin Li, Yanhua Liu, Feng Kong, Dawei Xu

**Affiliations:** 1 Central Research Laboratory, The Second Hospital, Cheeloo College of Medicine, Shandong University, Jinan, China; 2 Jinan Maternity and Child Health Care Hospital, Jinan, China; 3 Jinan Seventh People’s Hospital, Jinan, China; 4 Department of Central Laboratory, Shandong Provincial Hospital Affiliated to Shandong First Medical University, Jinan, China; 5 Engineering Laboratory of Urinary Organ and Functional Reconstruction of Shandong Province, Jinan, China; 6 Department of Medicine, Division of Hematology, Bioclinicum, Karolinska Institutet, Karolinska University Hospital Solna, Stockholm, Sweden

**Keywords:** Maternal infection, Newborns, Placental senescence, SARS-CoV-2, Telomere length

## Abstract

**Background:**

Initial telomere length (TL) in newborns is the major determinant for TL in later life while TL in newborn/early-life predicts long-term health and lifespan. It is important to identify key factors that affect telomere homeostasis throughout embryonic development for precision interventions to maintain optimal TL in fetus/prenatal infants. SARS-CoV-2 has caused a widespread global pandemic of COVID-19, but it remains unclear whether maternal SARS-CoV-2 infection impairs prenatal telomere homeostasis.

**Methods:**

We recruited 413 normally delivered newborns whose mothers were either non-infected or infected with SARS-CoV-2 during different trimesters of pregnancy (otherwise healthy). Telomere length (TL) in cord blood (CB) was assessed using qPCR. CB and maternal blood were analyzed for cytokine levels. Placental senescence was determined using senescence-associated β-galactosidase staining.

**Results:**

Control (non-infected maternal) newborn TL was significantly longer than that from maternal infection (1.568 ± 0.340 vs 1.390 ± 0.350, *P* = 0.005). Such shorter TL was observed only if maternal infection of SARS-CoV-2 occurred in the first and second trimesters of pregnancy (1.261 ± 0.340 and 1.346 ± 0.353, *P* < 0.0001 and 0.001, respectively). There were no differences in TL between controls and infection at the third trimester (1.568 ± 0.340 vs 1.565 ± 0.329, *P* > 0.05). Across the first trimester, there was a positive correlation between newborn TL and gestational weeks with maternal infection, suggesting that the earlier maternal infection occurs, the worse effect is taken on fetal telomere homeostasis. Placental senescence coupled with the downregulated expression of telomerase reverse transcriptase was significantly more frequent from the maternal infection at the first trimester. There were no differences in IL-6, C reactive protein and other cytokine levels in CB and maternal serum or placentas.

**Conclusions:**

Maternal SARS-CoV-2 infection at the first and second trimesters leads to significantly shorter TL and earlier infection causes much more severe TL damage. The infection-mediated cell senescence and other histopathological abnormalities result in defective placental function through which fetal telomere homeostasis is impaired. Thus, vaccination against COVID-19 should be done in advance for women who plan pregnancy.

**Supplementary Information:**

The online version contains supplementary material available at 10.1186/s12967-024-05879-0.

## Background

Human telomeres are the nucleoprotein complex consisting of 7–15 kb tandemly repeated TTAGGG sequences bound by 6 shelterin proteins, and they form a protective cap on chromosome ends essential to genomic stability/integrity [1, 2]. In cultured human cells, telomeres become progressively attrited with cellular divisions due to the end replication problem and genomic insults [1, 2]. When such telomere attrition reaches a threshold size (dysfunctional), the DNA damage response cascade is activated, and cells are subsequently triggered to enter a permanent growth arrest stage named “senescence” and/or apoptosis [1, 2]. In accordance with in vitro proliferative cells, telomeres shorten in vivo with increased age, which leads to accumulation of senescent cells, eventually resulting in functional decline in organs and tissues of old individuals [1, 2]. For instance, short telomeres induce immunosenescence, which is a key mechanism underlying diminished immune response to infection of various pathogens in the elderly [3, 4]. It has been shown that shorter leukocyte telomere length (TL) is strongly associated with age-related diseases including cardiovascular diseases, cancer, and among others, in adults [1]. Therefore, TL has long been used as a biomarker for aging and longevity [1]. Importantly, the evidence has emerged that initial TL in newborn infants is the major determinant for TL in later life, although environmental and other factors also increase TL attrition rates throughout adulthood [5–9]. Moreover, TL in early-life predicts long-term health and lifespan [6]. Thus, interventions to maintain optimal TL in prenatal and infants has been proposed for the healthy age across the one’s life course [8, 10, 11]. To this end, it is important to identify key factors that affect TL throughout embryonic development or in early life.

SARS-CoV-2, an RNA virus, has caused a widespread global pandemic of COVID-19 in the last years [12]. In most patients, the infection appears as asymptomatic disease or presents mild symptoms, however, a fraction of individuals, especially elderly people, are at high risk to develop severe COVID-19 with substantial mortality rates [12]. Moreover, sequelae of COVID-19 occur beyond the acute disease phase [13, 14]. A recent analysis of 153,760 individuals with COVID-19 showed that the risk and 1-year burden of cardiovascular disease in survivors of acute COVID-19 were significantly high, even among those who were not hospitalized during the acute phase of the infection [14]. By studying the mechanisms underlying severe diseases and late sequelae, several reports have shown their association with shorter TL and cellular senescence, which provides an explanation why the elderly are severe COVID-19-prone [4, 15–18]. On the other hand, SARS-CoV-2 infection accelerates telomere erosion and aging process and evokes virus-induced senescence (VIS) [15, 16, 19, 20]. Thus, shorter TL/senescence and SARS-CoV-2 infection impact each other to promote deteriorative situations. However, all the above studies are performed on adults, and it is currently unclear whether maternal SARS-CoV-2 infection has any effects on newborn TL. Moreover, because angiotensin-converting enzyme 2 (ACE2) receptors are strongly expressed in the syncytiotrophoblast from early pregnancy, SARS-CoV-2 is capable of infecting placentas directly, through which a vertical transmission may occur, too [21, 22]. Given all the facts/observations above, elucidating whether and how SARS-CoV-2 infection affect newborn TL has biological and clinical implications. The present study is thus designed to address this issue.

## Methods

### Study population

Pregnant women who received prenatal care and delivered normally at Jinan Maternity and Child Health Care Hospital and Shandong Provincial Hospital were recruited between Dec. 2022 and Oct. 2023. None of them were active or passive smokers, consumed alcohol daily, and had medical history of diabetes, heart, kidney, liver or other chronic diseases. Moreover, in all the recruited women, their spouses were not infected before pregnancy. A total of 413 participants were included in the study and 380 of them were infected by SARS-CoV-2 during three different trimester periods (first, n = 129; second, n = 123 and third, n = 128), as diagnosed using PCR assays of nasopharyngeal swab [Novel Coronavirus (2019-nCoV) Dual Probes qRT-PCR Kit, Shanghai BioGerm Medical Technology Co., Ltd.]. Among infected women, 53 of 380 were asymptomatic, while remaining 327 had fever [37.3–38 °C (n = 96), 38.1–39 °C (n = 219) and > 39 °C (n = 12)] and other symptoms. Thirty-three non-infected pregnant women were recruited as healthy controls. Placental tissues were obtained from 109 participants. The study was approved by Shandong Provincial Hospital Ethics Committee (#2022-297) and informed consent was obtained from all the subjects.

### Cord blood (CB) collection and TL assessment

Five ml of umbilical CB were collected from each newborn infant. We excluded subjects if gestational age < 32 weeks, birth weight < 1500 g, or the presence of any congenital diseases. Genomic DNA was extracted using a TIANGEN TIANamp Genomic DNA kit (Tiangen Biotech (Beijing) Co., Ltd; Beijing, China). TL assessment was described previously [23, 24]. Briefly, 10 ng of DNA were used for each PCR reaction in triplicate. The primers and their sequences used in the present study are listed in Table S1. *T*/HBG (*S*) values (*T*: telomere repeat copy number, and *S*: single-copy gene number HBG) were calculated using the formula *T*/*S* = 2 − ΔCt, where 2ΔCt = average Ct_telomere_ − average Ct_β-globin_. The *T*/*S* ratio was arbitrarily expressed as telomere length.

### ELISA assay of IL-6 and C reactive protein (CRP)

Maternal and CB serum was analyzed for IL-6 and CRP levels using ELISA kits (Roche Cobas e analyzers Elecsys IL-6, #09015604, Roche Diagnostics Co., Ltd. Shanghai, China) and Super-sensitive CRP assessment kits (#F0311, Sichuan Xincheng Biological Co., Ltd, Chengdu, China), respectively.

### RNA extraction and qRT-PCR

Total RNA was extracted from cord blood cells and placenta tissues using Trizol-Reagent TRIzol Reagent (#267310, Ambion by life technologies™, Life technologies Corporation; Carlsbad). RNA was reversely transcribed using ReverTra Ace qPCR RT Master Mix (#FSQ-201, Toyobo Co., Ltd. Life Science Department Osaka Japan). qPCR was carried out using SYBR Green Realtime PCR Master Mix (Code QPK-20, Toyobo Co., Ltd). Target mRNA quantification was done based on the 2−ΔCT values and normalized to human GAPDH expression. Primers and their sequences are listed in Table S1.

### Senescence-associated β-galactosidase (SA-β-Gal) staining

Senescent cells in placenta were determined by SA-β-Gal staining using the Senescence β-Galactosidase Staining kit (Beyotime Biotechnology, Shanghai) according to the manufacturer's protocol. Briefly, Unfixed placenta slides were stained with X-gal solution (pH = 5.9–6.1) at 37 °C in a dry incubator for 24 h. Images were captured by digital phase contrast microscopy. The SA-βgal blue intensity for each image (in three different areas) was measured with ImageJ software (NIH), which were then averaged to represent the mean SA-βgal blue intensity per placenta. Positive and negative control samples were included in staining experiments.

### Statistical analysis

Differences in telomere length between different groups were assessed using Student T-test or Mann–Whitney U test dependent on distributions. The relationship between TL and other variables was assessed using Spearman test. Kruskal–Wallis test was used to evaluate differences in maternal parameters, prenatal Ultra-Sound examinations and placenta senescence among different groups. All the tests were computed using SPSS 26.0 or Graphpad Pism 9.5 software. *P* values of < 0.05 were considered statistically significant.

## Results

### Baseline characteristics of pregnant women and their newborns

A total of 413 pregnant women were recruited and 33 of them were non-infected healthy controls, while 380 were infected with SARS-CoV-2 and infection took place in the first (n = 129), second (n = 123) and third (n = 128) trimester, respectively. General characteristics of these pregnant women are documented in Table 1, and there were no significant differences between non-infected and infected mothers, and among groups infected in 3 different trimester periods.

**Table 1:** Summary of maternal and newborn information

	Uninfected	First-trimester	Second-trimester	Third-trimester	P-Value
	(N = 33)	(N = 129)	(N = 123)	(N = 128)	
	Mean ± SD	Mean ± SD	Mean ± SD	Mean ± SD	
Maternal age (year)	30.00 ± 4.18	29.31 ± 4.74	29.17 ± 4.16	30.09 ± 4.02	0.177
Spouse age (year)	31.17 ± 4.74	30.06 ± 4.43	30.12 ± 4.30	30.98 ± 4.44	0.228
Prepregnancy weight (kg)	58.32 ± 5.52	57.28 ± 7.36	59.27 ± 8.42	59.42 ± 11.09	0.244
Prepartum weight (kg)	73.36 ± 6.59	71.82 ± 8.42	74.48 ± 8.87	73.90 ± 11.71	0.150
Height (cm)	163.94 ± 5.44	162.78 ± 4.21	163.05 ± 5.56	162.89 ± 4.70	0.797
Prepregnancy BMI (kg/m^2^)	21.75 ± 2.36	21.62 ± 2.65	22.31 ± 3.16	22.35 ± 3.83	0.380
Prenatal BMI (kg/m^2^)	27.37 ± 2.92	27.11 ± 3.02	28.03 ± 3.22	27.81 ± 3.98	0.175
Abdominal circumference (cm)	102.31 ± 6.33	101.07 ± 6.86	102.44 ± 5.93	102.16 ± 7.10	0.289
Fundal height (cm)	34.31 ± 1.95	34.65 ± 2.77	34.30 ± 1.72	34.23 ± 2.06	0.665
White blood cell count (× 10^9^/l)	9.95 ± 2.65	9.38 ± 2.31	9.46 ± 2.27	9.12 ± 2.99	0.217
Percentage of neutrophil (%)	74.66 ± 4.92	74.28 ± 7.14	73.04 ± 7.35	71.87 ± 7.72	0.017
ALT (µ/l)	10.69 ± 4.40	11.05 ± 5.90	10.90 ± 5.19	13.77 ± 9.27	0.053
AST (µ/l)	14.13 ± 3.17	14.15 ± 3.34	15.05 ± 10.24	15.94 ± 6.25	0.165
Creatinine (µmol/l)	43.58 ± 6.23	45.80 ± 7.67	44.51 ± 6.95	45.05 ± 11.25	0.119
Urea nitrogen (mmol/l)	2.98 ± 0.73	3.09 ± 0.71	3.13 ± 0.79	4.52 ± 14.40	0.799
Uric acid (µmol/l)	250.23 ± 71.76	243.16 ± 58.29	239.07 ± 54.83	255.51 ± 67.66	0.133
Weight of the newborn baby (g)	3382.35 ± 501.34	3242.08 ± 342.38	3311.46 ± 433.84	3291.25 ± 467.33	0.193
Gravidity	1.91 ± 0.92	1.87 ± 1.01	1.82 ± 0.94	2.08 ± 1.12	0.294
Parity	1.46 ± 0.56	1.44 ± 0.60	1.46 ± 0.62	1.47 ± 0.56	0.895
Sex of the newborn baby					0.344
Male	16 (48%^a^)	55 (43%^a^)	61 (50%^a^)	69 (54%^a^)	
Female	17 (53%^a^)	74 (57%^a^)	62 (50%^a^)	59 (46%^a^)	

*BMI* body mass index, *ALT* Alanine transaminase, *AST* Aspartate transaminase, *SD* standard deviation

^a^Represents the proportion of different sexes in each group

Table S2 lists newborn characteristics and their final prenatal Ultra-sound examination. There were no differences among the different groups.

### Analysis of SARS-CoV-2 presence in placentas

To determine whether SARS-CoV-2 was present in placentas, we analyzed a total of 109 placentas, both chorion and decidua. As expected, all 20 placentas from non-infected mothers were negative for SARS-CoV-2, while the total positivity was 33.33% and 6.06% for infection in the third and second trimester, respectively; and the virus was present on either chorion or decidua or both (Table S3). All the placentas from infection in the first trimester were SARS-CoV-19 negative except one with repeated infection during third trimester (Table S3).

### Impacts of maternal SARS-CoV-2 infection on newborn TL in trimester-dependent manners

**Fig. 1:**
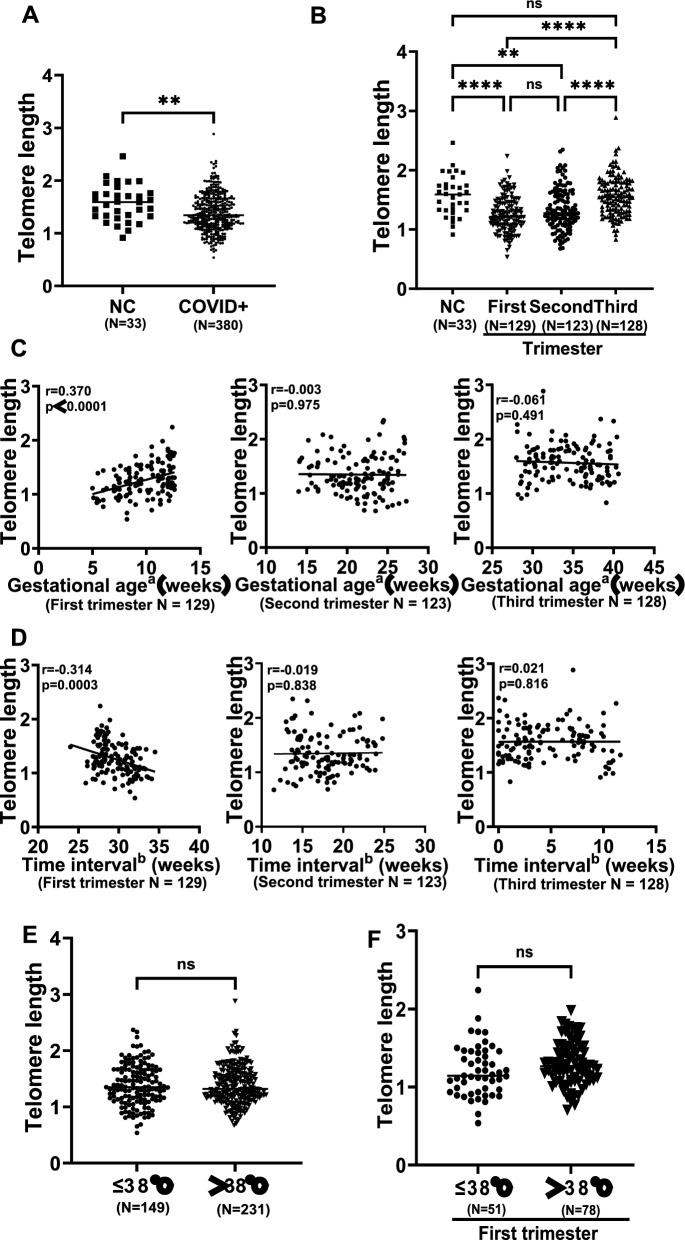
Maternal SARS-CoV-2 infection in the first and second trimester causes telomere length (TL) shortening in newborns. Newborn TL in cord blood cells were assessed using qPCR. **A** Significantly shorter TL in newborns from maternal SARS-CoV-2 infection. **B** TL shortening in newborns from maternal infection in the first and second but not third trimester. **C** A positive correlation between newborn TL and gestational age of infection in the first trimester. Gestational age^a^, gestational age when maternal infection occurs. **D** An inverse correlation between newborn TL and time interval from infection in the first trimester to delivery. Time interval^b^, time interval from infection to delivery. **E**, **F** No association between newborn TL shortening and COVID-19 severity in all newborns (**E**) or infection in the first trimester (F). NS, not significant. *, **, *** and ****, *P* < 0.05, 0.01, 0.001 and 0.0001, respectively

Newborn TL was represented by that in CB cells, as assessed using qPCR. Newborns were first categorized into the following two groups: with and without maternal SARS-CoV-2 and infection. As shown in Fig. 1A, significantly shorter TL occurred in newborns whose mothers were infected with SARS-CoV-2 (noninfected vs infected: 1.568 ± 0.340 vs 1.390 ± 0.350, *P* = 0.005). We then sought to determine whether maternal infection in the different trimesters had differential effects on newborn TL. Offsprings carried the shortest TL when maternal infection occurred in the first trimester (1.261 ± 0.300, *P* < 0.0001 compared to infected in the third trimester, 1.565 ± 0.329), which was followed by the infection in the second trimester (1.346 ± 0.353, *P* < 0.0001 compared to infected in the third trimester, 1.565 ± 0.329) (Fig. 1B). TL was comparable between newborns from noninfected mothers and those infected in the third trimester (1.568 ± 0.340 vs 1.565 ± 0.329, *P* > 0.05) (Fig. 1B). These observations suggest that the newborn TL was negatively impacted when maternal infection occurs in the first and second trimester. Moreover, in the first trimester infection, newborn TL was correlated positively with gestational age, but negatively with the time interval between infection and delivery (Fig. 1C, D). Such correlations were not observed in those whose maternal infection occurred in the second and third trimester (Fig. 1C, D). We also analyzed the association between newborn TL and mothers’ COVID-19 severity and simply divided them into two groups with asymptomatic/low fever (37.3–38 °C) and middle-high fever (> 38 °C). The TL comparison of all newborns showed no differences between two groups (1.39 ± 0.36 vs 1.39 ± 0.35, *P* = 0.979) (Fig. 1E). Because TL was shortest in newborns from maternal infection in the first trimester, they were further analyzed separately, and TL did not differ between ≤ 38 °C and > 38 °C groups (1.21 ± 0.33 vs 1.30 ± 0.28, *P* = 0.092) (Fig. 1F).

**Fig. 2:**
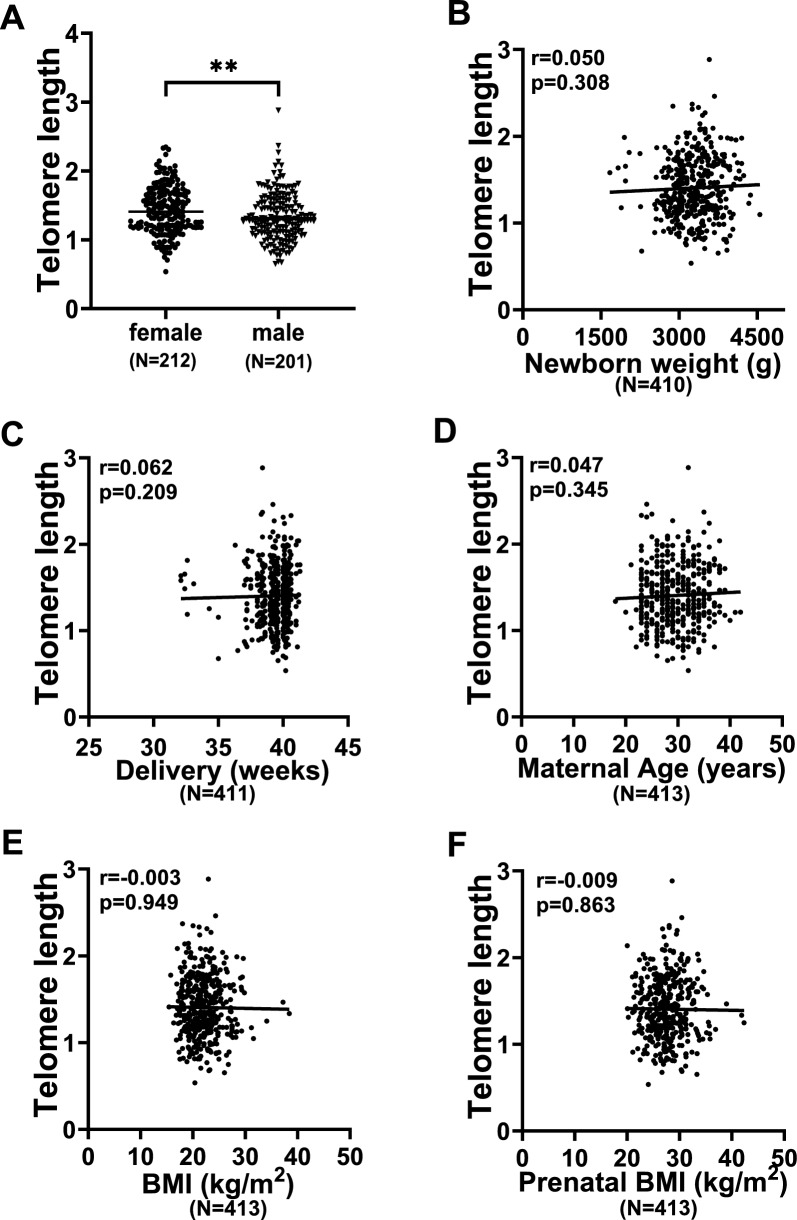
Differences or correlation of newborn TL with neonate sex, weight, gestational age and maternal BMI. **A** Significantly longer TL in female newborns than male ones. **B** Lack of correlation between newborn TL and weight. **C** No correlation between newborn TL and gestational age. **D** No correlation between newborn TL and maternal age. **E**, **F** No correlation between newborn TL and pre-pregnant or pre-delivery BMI. **, *P* < 0.01

TL is in general longer in female infants than in male ones [25]. Consistent with early observations, female newborns carried significantly longer TL than did male ones (Fig. 2A) (1.45 ± 0.35 vs 1.36 ± 0.35, *P* = 0.004). In addition, we further determined the potential impact of mother’s age at delivery, pre-pregnancy BMI, prenatal BMI, infant weight and gestational age on newborn TL. As shown in Fig. 2B–F, these variables had no significant association with newborn TL, which is in accordance with non-differences in maternal and newborn characteristics between different groups, as shown in Table 1 and Table S2.

### Analyses of inflammatory factors and cytokines in serum between infected and non-infected groups

**Fig. 3:**
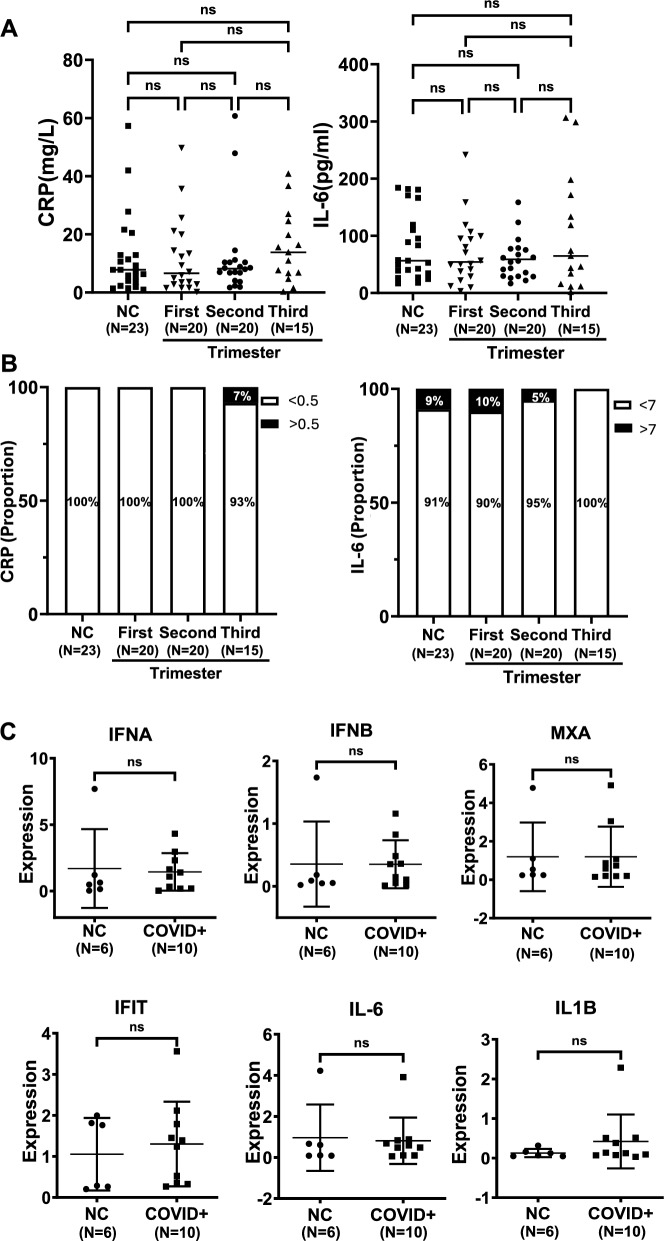
Expression of inflammatory factors and cytokines in serum from maternal blood and cord blood, or placentas. CRP and IL-6 protein levels in serum were assessed using ELISA. **A** No differences in serum CRP and IL-6 levels among groups without and with maternal SARS-CoV-2 infection in different trimesters. NS, not significant. **B** No differences in cord blood CRP and IL-6 levels among newborn groups without and with maternal SARS-CoV-2 infection in different trimesters. For CRP and IL-6, detectable threshold levels were 0.5 mg/L and 7 pg/L, respectively. **C** No differences in IFNA, IFNB, MXA, IFIT, IL-6 and IL-1B expression between placentas without and with maternal SARS-CoV-2 infection. The assessment was done using qPCR. IFNA and IFNB, Interferon A and B; MXA, Myxovirus-resistant protein 1; Interferon induced protein with tetratricopeptide repeats, IFIT; Interleukin 1 beta (IL-1B)

Because SARS-CoV-2 infection-induced cytokine storm plays a key role in the COVID-19 pathogenesis, we compared differences in CRP and IL-6 levels in serum derived from both CB and maternal blood. As shown in Fig. 3A, CRP and IL-6 levels in maternal serum were largely similar among all the analyzed groups. CB serum from 78 neonates were examined and they consisted of those with non-infected mothers (n = 23), and mothers infected in the first (n = 20), second (n = 20) and third (n = 15) trimester. CRP (> 0.5 mg/ml) was only detectable in one neonate (1/78) (mother infected in the third trimester), while IL-6 (> 7 pg/ml) was found in 5 of them (6.4%) (Fig. 3B). Moreover, we examined placenta for interferon A and B (IFNA and IFNB), myxovirus-resistant protein 1 (MXA), interferon induced protein with tetratricopeptide repeats (IFIT), IL-6 and interleukin 1 beta (IL1B) expression using qPCR and observed comparable levels of these mRNAs among different groups (Fig. 3C).

### Placental senescence and TERT downregulation resulting from the maternal infection

**Fig. 4:**
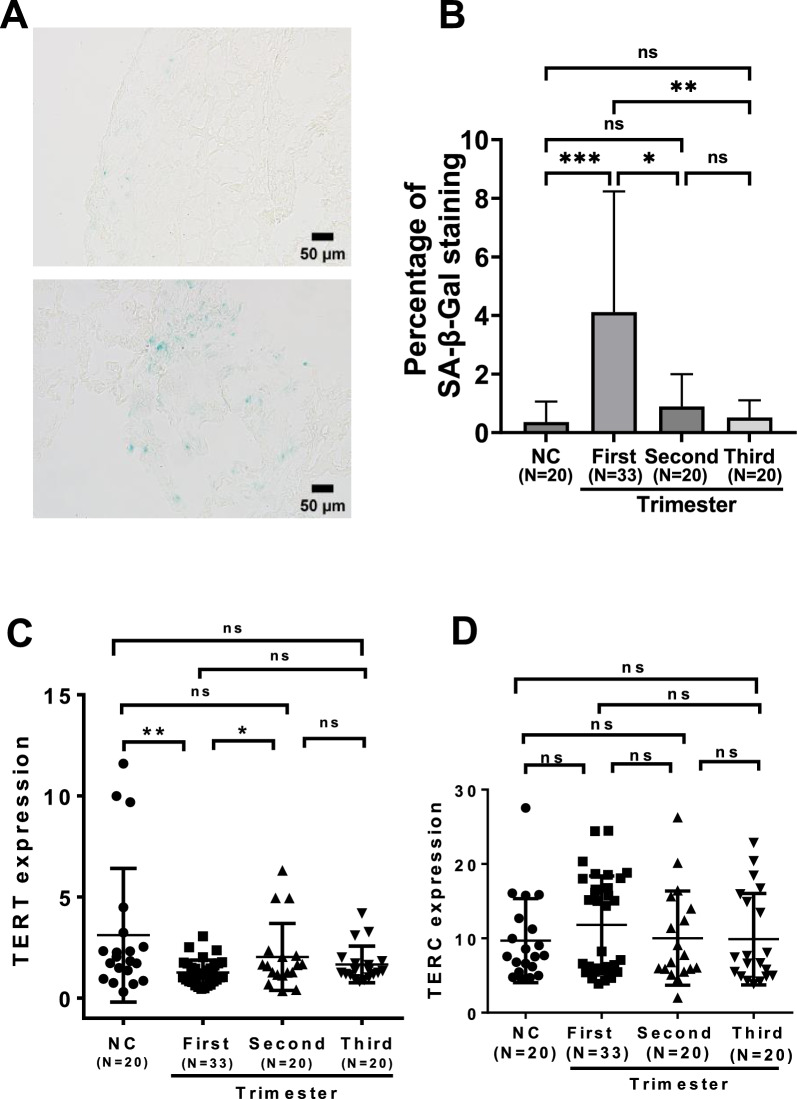
Placental senescence and TERT downregulation mediated by maternal SARS-CoV-2 infection in the first trimester. Senescence-associated β-galactosidase (SA-β-Gal) staining was used to assess placental senescence from different groups. TERT and TERC expressions were determined using qRT-PCR. **A** Representative images from the non-infected (Top) and infected (Bottom) placentas. **B** Quantification of positive SA-β-Gal staining cells. **C** TERT mRNA levels in the placentas from non-infected women and those infected at different trimesters, as determined using qRT-PCR. **D** TERC RNA levels in the placentas from non-infected women and those infected at different trimesters, as determined using qRT-PCR. *, ** and ***, *P* < 0.05, 0.01 and 0.001, respectively. ns, not significant

SARS-CoV-2 infection induces VIS, while we detected its presence in placentas from maternal infection. Therefore, we sought to determine whether placental senescence occurred due to maternal infection. SA-β-Gal staining was employed to examine the presence of senescent cells in the placenta. A total of 93 placentas were included, among which were 20, 33, 20, and 20 from non-infected, and maternal infection in the first, second and third trimester, respectively. The placentas from the first trimester infection contained significantly higher SA-β-Gal -positive cells than any other groups (4.11% ± 4.13%, *P* = 0.0001), whereas non-infected and infected in the second and third trimester were 0.36% ± 0.70%, 0.89% ± 1.10%, 0.52% ± 0.59%, respectively (Fig. 4A, B).

TERT, as the rate-limiting component for telomerase activity, is required for lengthening telomeres and preventing senescence, and thus, we further determined TERT mRNA expression in the placentas above. Relative levels of TERT mRNA for non-infected, and maternal infection in the first-, second- and third-trimester were 3.11 ± 0.74, 1.27 ± 0.11, 2.04 ± 0.39 and 1.67 ± 0.21, respectively (Fig. 4C). The difference was highly significant between non-infected women and those with infection at the first trimester (*P* = 0.003), while there were no differences when non-infected women were compared with infected ones occurring in the second and third trimesters (Fig. 4C). We also analyzed the level of TERC, a ubiquitously expressed telomerase RNA component, and did not observe significant differences among those 4 groups (9.68 ± 1.26, 11.81 ± 1.16, 10.02 ± 1.49 and 9.89 ± 1.38, for non-infected, and infected in the first, second and third trimester, respectively) (Fig. 4D).

## Discussion

In the present study, we evaluated the association between maternal SARS-CoV-2 infection and newborn TL. Because smoking, obesity, parental age and preterm birth all influence newborn TL [9, 10, 26], we controlled these variables and excluded their potential interferences while recruited. Our results showed that the infection in the first and second trimester of pregnant women led to significantly shortened TL in newborn infants, but no impacts on TL if the infection occurred in the third trimester. Across the first trimester, there was a positive correlation between newborn TL and gestational weeks with maternal infection, while a negative correlation between newborn TL and time to delivery. These findings indicate that telomere homeostasis in the very early period of embryonic development is extremely sensitive to SARS-CoV-2 infection.

It is currently unclear how maternal SARS-CoV-2 infection contributes to newborn TL loss. Vertical transmission of SARS-CoV-2 is not a frequent event due to the barrier function of the placental syncytiotrophoblast layer [27], and therefore, the observed effect may be indirect. Because the infection-induced cytokine storm is a key mechanism underlying the COVID-19 pathogenesis [20], we compared IL-6 and CRP levels in offspring CB between uninfected and infected mothers, but there were no significant differences. These results are not unexpectable, as in the vast majority of pregnant women, the infection occurred in long before delivery. As described above, SARS-CoV-2 infection induces senescence of infected cells so-called VIS, and senescent cells play an important role in the COVID-19 pathogenesis [15, 16]. Moreover, ACE2 receptors are strongly expressed in the syncytiotrophoblast from early pregnancy [21], and SARS-CoV-2 is capable of infecting placenta directly, as shown by the present and other studies [21, 22]. Because cell senescence is in general irreversible and persists after the initial infection with SARS-CoV-2, we determined whether senescent cells were presented in placenta from infected mothers. Indeed, senescent cells were detected in the placenta from fractions of infected mothers, and intriguingly, almost all positive samples were from those when the infection took place in their first trimester. We further observed that TERT expression level was lowest in the placentas from women infected in the first trimester. It is thus likely that the downregulated TERT fails to compensate for TL loss, further promoting senescence. Senescent cells are expected to impair placental function, thereby exerting negative effects on TL homeostasis. Consistently, TL loss was severest in their offsprings. These findings support the concept that newborn TL loss is attributable to placental cell senescence induced by SARS-CoV-2.

In addition to senescence identified in the present study, SARS-CoV-2 infection has been shown to induce multiple histopathological abnormalities of placenta in pregnant women, including maternal/fetal vascular malperfusion, acute and chronic inflammatory pathologies, increased perivillous fibrin, histiocytic intervillositis and trophoblast necrosis, and among others [21, 22]. All these lesions collectively lead to placental dysfunction and hypoperfusion and fetal hypoxia, thereby impairing fetal development/growth or even stillbirth [22]. In that case, damaged TL homeostasis is unavoidable. Intriguingly, placental lesions similarly occur even if pregnant women are with asymptotic infection [21]. These findings are in accordance with our observation that neonate TL loss is independent of asymptomatic and symptomatic maternal infection.

Our findings show that newborn TL becomes significantly lost when infection occurs in the first and second trimester, and moreover, longer interval from the infection to delivery results in more severe TL shortening in the first trimester. The mechanism underlying this phenome is unclear. A putative explanation is that placental development is not complete during the first trimester, which is more vulnerable to the insults mediated by SARS-CoV-2 infection. We previously found that there was a sharp reduction followed by a slow attrition of TL in human fetus from gestational age 6–11 weeks within the first trimester, while telomerase activity response for telomere extension was steadily downregulated during this same period [28]. Likely, maternal infection further accelerates fetal TL erosion or interferes with telomere lengthening in this critical interval. Further studies are required to elucidate this issue.

Newborn TL is affected by an adverse intrauterine environment resulting from various maternal and outside factors [9, 26, 29]. Evidence has accumulated that the reduced TL in the newborn setting has negative effects on long-term health or lifespan [5–9, 11]. For instance, shorter telomeres in early childhood have a significantly higher frequency to develop arterial wall thickness and hypertension later in life, which suggests an association between relatively short telomeres in early childhood and vascular disease risk later in life [11, 30]. Therefore, identifying the factors that are detrimental to prenatal TL and avoiding maternal exposure to them should result in optimal TL homeostasis in infants and consequent healthy aging in late life. Based on our present findings, it is necessary to pay attention to the short and long-term health in offsprings of mothers who are infected with SARS-CoV-2 during their first and second trimesters of pregnancy.

The present study has limitations. We only included 33 newborns whose mothers were non-infected during the pregnancy period, and the sample size of the control group was small. The pandemic control policy change in the end of 2022 resulted in a huge-scale epidemic outbreak, causing infection of almost all the population in China, and it was difficult to recruit more non-infected pregnant women and their newborns. Thereafter, pregnant women without infection were available, but their spouses were exclusively infected before pregnancy. It has been shown that adult males exhibited reduced semen volume, sperm motility and the progressive sperm motility rate after SARS-CoV-2 infection [31], which may lead to unpredictable consequences. We are currently preparing to address whether the infected males undergo TL alterations in their sperm cells or spermatocytes, whether paternal infection induces TL shortening in the offsprings, too, and if so, how long will take to recover, or when it is suitable time for them to have offsprings after infection. Nevertheless, it is evident from the present study that the newborns have comparable TL between maternal infection in the third trimester and non-infected controls, indicating that the findings based on the small size of control samples are reliable.

## Conclusions

We show that newborn TL is significantly shorter if maternal infection of SARS-CoV-2 occurs in the first and second trimesters of pregnancy. Likely, the infection-mediated cell senescence and other histopathological abnormalities lead to defective placental function through which fetal telomere homeostasis is impaired. Given the association between newborn TL and overall health and future aging-related diseases, vaccination against COVID-19 should be done in advance for women who plan pregnancy to maintain optimal TL in prenatal and infants.

## Supplementary Information

Supplementary Material 1: Table S1. Primers and sequences used in the present study. Table S2. Final Ultra-sound examination of newborns. Table S3. Placental detection of SARS-CoV-2.

## Data Availability

All the data is included in the manuscript.

## Abbreviations

ACE2: Angiotensin-converting enzyme 2
BMI: Body mass index
CB: Cord blood
CRP: C reactive protein
IFIT: Interferon induced protein with tetratricopeptide repeats
IFNA: Interferon A
IFNB: Interferon B
IL-1B: Interleukin 1 beta
MXA: Myxovirus-resistant protein 1
SA-β-Gal: Senescence-associated β-galactosidase
TL: Telomere length
VIS: Virus-induced senescence
