# Supplementary material for: Maternal infection of SARS-CoV-2 during the first and second trimesters leads to newborn telomere shortening
Source: J Transl Med. 2024 Nov 21;22:1049. doi: 10.1186/s12967-024-05879-0 (PMC11580642; doi:10.1186/s12967-024-05879-0)
Supplement: Supplementary file 1 — Supplementary Material 1: Table S1. Primers and sequences used in the present study. Table S2. Final Ultra-sound examination of newborns. Table S3. Placental detection of SARS-CoV-2. [file 12967_2024_5879_MOESM1_ESM.pdf]

Table S1. Primers and sequences used in the present study

Gene	primers
IFNA2-F	GCTTGGGATGAGACCCTCCTA
IFNA2-R	CCCACCCCCTGTATCACAC
IFNB-F	GCTTGGATTCCTACAAAGAAGCA
IFNB-R	ATAGATGGTCAATGCGGCGTC
MXA-F	GGTGGTCCCCAGTAATGTGG
MXA-R	CGTCAAGATTCCGATGGTCCT
IL6-F	ACTCACCTCTTCAGAACGAATTG
IL6-R	CCATCTTTGGAAGGTTCAGGTTG
IL1B-F	AGCTACGAATCTCCGACCAC
IL1B-R	CGTTATCCCATGTGTCGAAGAA
IFIT1-F	AGAAGCAGGCAATCACAGAAAA
IFIT1-R	CTGAAACCGACCATAGTGGAAAT
GAPDH-F	GGAGCGAGATCCCTCCAAAAT
GAPDH-R	GGCTGTTGTCATACTTCTCATGG
TEL-F	CGGTTTGTTTGGGTTTGGGTTTGGGTTTGGGTTTGGGTT
TEL-R	GGCTTGCCTTACCCTTACCCTTACCCTTACCCTTACCCT
HBG-F	TGTGCTGGCCCATCACTTTG
HBG-R	ACCAGCCACCACTTTCTGATAGG

Table S2. Ultra-sound examination of newborns before delivery

	Uninfected (N=33)	first-trimester (N=106)	second-trimester (N=120)	third-trimester (N=123)	P-Value
	$\bar{x} \pm s$	$\bar{x} \pm s$	$\bar{x} \pm s$	$\bar{x} \pm s$	
Biparietal diameter（mm)	94.17±4.26	93.30±3.55	93.09±4.10	92.70±4.70	0.515
Head circumference (mm)	331.31±15.82	332.15±10.17	329.31±30.04	328.02±30.36	0.920
Abdominal circumference（mm）	341.66±20.00	339.87±16.56	341.88±18.85	339.68±22.34	0.514
Femur length（mm）	71.00±3.33	71.19±2.90	76.06±53.48	71.07±3.95	0.876
Umbilical blood flow（S/D)	2.29±0.33	2.30±0.31	2.29±0.28	2.28±0.28	0.993
Fetal heart rate	144.09±10.28	141.79±11.64	141.13±11.80	144.33±11.04	0.105
Amniotic fluid index（/min)	113.51±37.03	118.66±30.92	118.23±36.54	121.02±38.69	0.786

Table S3. Placental detection of SARS-CoV-2

Infection time (trimester)	Numbers		Chorion positive	Decidua positive	Both positive	Positivity (%)
	Chorion	Decidua				
First	20	20	0	1*	0	3.84%
Second	20	20	1	1	0	6.06%
Third	20	20	7	3	2	33.33%
Non-infected	20	20	0	0	0	0

*Infected during both first and third trimesters.
